# A Rare Case of Isolated Abducens Nerve Palsy With Contemporaneous Thalamic Infarct

**DOI:** 10.7759/cureus.56268

**Published:** 2024-03-16

**Authors:** Lai Zhong Yang, Qi Zhe Ngoo, Shanti Viswanathan, Rafidah Sudarno

**Affiliations:** 1 Department of Ophthalmology and Visual Science, School of Medical Sciences, Universiti Sains Malaysia, Kota Bharu, MYS; 2 Department of Ophthalmology, Universiti Sains Malaysia, Kota Bharu, MYS; 3 Department of Neurology, Hospital Kuala Lumpur, Kuala Lumpur, MYS; 4 Department of Ophthalmology, Hospital Tengku Ampuan Rahimah, Klang, MYS

**Keywords:** hemiparesis, ptosis, ophthalmoplegia, isolated abducens nerve palsy, internal capsule infarct, thalamic infarct

## Abstract

We report a case of isolated left abducens nerve palsy accompanying a right thalamic infarct. The patient, a 43-year-old Malay male with newly diagnosed hypertension, diabetes mellitus, and dyslipidemia, initially reported binocular diplopia on left lateral gaze persisting for five weeks. Subsequently, he experienced acute left-sided body weakness and slurred speech for over one day. Clinical examination revealed restricted left eye lateral gaze (-3) with no relative afferent pupillary defect. Additionally, decreased power (4/5) was noted in the left upper and lower limbs. Brain magnetic resonance imaging (MRI) revealed restricted diffusion in the right thalamus extending to the right posterior internal capsule, left anterior cingulate gyrus, and left caudate nucleus. The patient was initiated on antiplatelet, antihypertensive, and oral hypoglycemic agents, resulting in symptom improvement. This rare neuroophthalmological finding has not been reported previously.

## Introduction

The abducens nerve, also referred to as the sixth cranial nerve, innervates the lateral rectus muscle of the same side, which is responsible for moving the eye outward (abduction). It originates from the abducens nucleus located in the lower dorsal pons. Exiting the nucleus anteriorly, it forms the abducens fascicle and then exits the brainstem at the junction between the pons and the medulla oblongata, entering the subarachnoid space [1]. Upon making an acute angle turn, the nerve sharply ascends over the clivus and the petrous apex, passing below the petroclival ligament and piercing Dorello’s canal, where it is vulnerable to increased intracranial pressure and trauma due to dural tethering [2,3]. Continuing its course, it traverses the cavernous sinus alongside the internal carotid artery, before passing through the superior orbital fissure to innervate the lateral rectus muscle [4]. Abducens nerve palsy represents the most common ocular motor paralysis in adults, with common etiologies including infarction, tumor, inflammation, meningitis, trauma, cavernous sinus lesions, and orbital apex syndrome.

Anatomically, the human thalamus comprises a constellation of nuclei situated in the diencephalon, including the hypothalamus, the epithalamus, the prethalamus, and the dorsal thalamus [5]. It functions as a hub for relaying both sensory and motor signals, with around 50-60 thalamic nuclei sending signals to specific cortical regions [6]. In terms of function, the thalamic nuclear complex can be divided into five primary functional categories as follows: effector nuclei, playing roles in motor function and language; sensory nuclei, involved in all major sensory domains; reticular and intralaminar nuclei, responsible for arousal and nociception; limbic nuclei, associated with mood and motivation; and associative nuclei, contributing to high-level cognitive functions [7]. Thalamic afferents and efferents typically exhibit contralateral organization, although some nuclei have bilateral connections, and thalamic functions affect both sensory and motor mechanisms. The thalamus is primarily supplied with blood from four arterial branches that stem from the posterior communicating artery (PCoA) and the initial segments of the posterior cerebral arteries (PCA). Vascular incidents impacting the thalamus, like infarction or hemorrhage, can result in somatosensory abnormalities and/or central pain on the opposite side of the body, leading to a thalamic syndrome characterized by contralateral numbness (or hypoesthesia), contralateral weakness, lack of coordination, and enduring spontaneous pain [8-10].

## Case presentation

**Figure 1:**
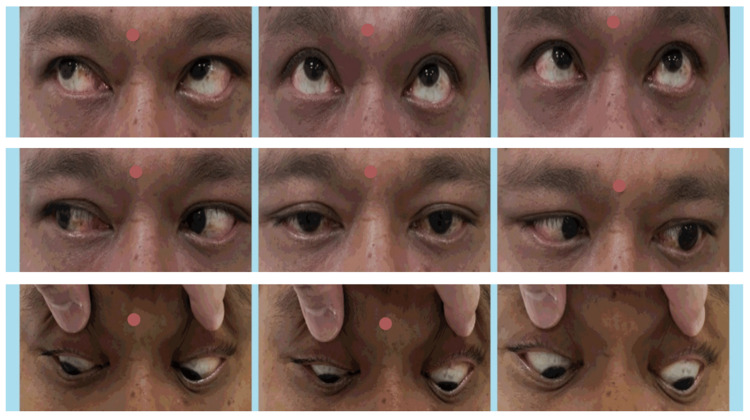
Left eye lateral gaze restriction and right eye partial ptosis.

A 43-year-old Malay male, recently diagnosed with hypertension, diabetes mellitus, and dyslipidemia, presented with sudden onset binocular diplopia upon left lateral gaze persisting for five weeks. He subsequently developed acute left-sided body weakness and slurred speech for more than one day. Examination revealed a restriction of -3 in left eye lateral gaze (Figure 1).

Additionally, no relative afferent pupillary defect was noted. For the ocular examinations, the best corrected visual acuity for both eyes was recorded as 6/6, with an intraocular pressure of 16 mmHg. Anterior segment-wise findings for both eyes included non-injected conjunctiva, clear cornea, deep and quiet anterior chamber, round and reactive pupils measuring 3 mm in size, and clear lenses. Moving to the posterior segments, both eyes exhibited a pink optic disc with a cup-disc ratio of 0.3, normal macula, and flat retina.

Furthermore, there was a reduction in power to 4/5 in both the left upper and lower limbs. Initial random blood sugar was recorded at 20.8 mmol/L, with a blood pressure of 178/94 mmHg. Table 1 outlines the results of the initial blood investigations.

**Table 1:** Initial blood investigations. HDL: high-density lipoprotein

Blood investigations	Results	Normal range
HbA1c	8.8%	<5.7%
HDL cholesterol	0.9 mmol/L	≥1.6 mmol/L

**Figure 2:**
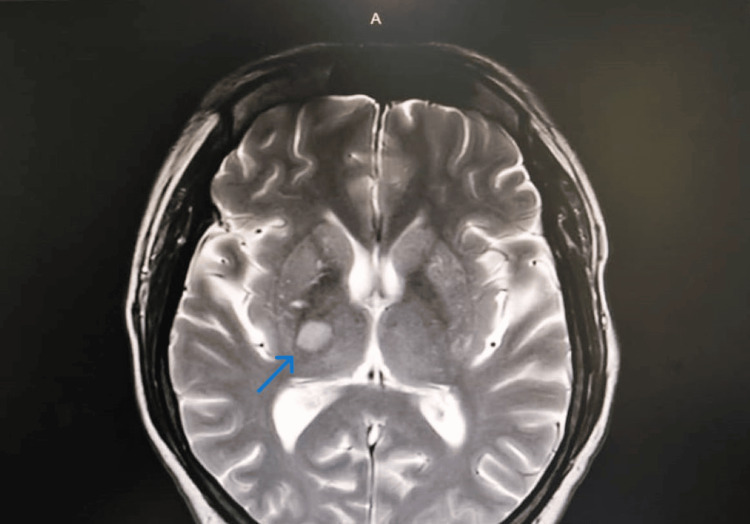
MRI shows restricted diffusion to right thalamus and posterior internal capsule (arrow).

The initial computed tomography (CT) scan of the brain revealed no recent infarct or hemorrhage. However, magnetic resonance imaging (MRI) of the brain showed restricted diffusion in the right thalamus extending to the right posterior internal capsule, as well as involving the left anterior cingulate gyrus and left caudate nucleus (Figure 2).

The imaging also revealed an old infarct in the left corona radiata and right middle cerebellar peduncle, extending to the right cerebellar hemisphere, along with minimal intracranial atherosclerotic disease involving the left cavernous internal carotid artery and right middle cerebral artery. No aneurysmal dilatation or vascular malformation was observed, and there was no evidence of midline shift or hydrocephalus. The patient underwent a multi-disciplinary approach to management, involving several teams including ophthalmology for eye care, neuromedicine for stroke management, radiology for precise imaging, and physiotherapy and rehabilitation for early mobilization and functional improvement. Treatment included Tab. aspirin 150 mg once daily, Tab. amlodipine 10 mg once daily, Tab. metformin 1 g twice daily, and Tab. atorvastatin 20 mg once nightly. The patient responded well to treatment with rapid recovery noted.

During the follow-up after discharge, his fasting blood sugar measured 5.0 mmol/L with a blood pressure reading of 136/78 mmHg. Improvement was observed in his left abducens nerve palsy, evidenced by the left lateral gaze at -1, with no diplopia detected. Additionally, the patient displayed the capacity to walk and engage in regular activities.

## Discussion

In this case, the patient experienced an acute episode of left abducens nerve palsy, followed by a rather uncommon occurrence of a right thalamic and internal capsule stroke, alongside evidence of an old infarct in the right middle cerebellar peduncle and hemisphere.

It's uncommon for isolated abducens nerve palsy to coincide with a thalamic infarct. However, there have been reports of other rare neuroophthalmological presentations. For example, a case report details a distinctive vertical one-and-a-half syndrome accompanied by a pseudo-abducens palsy on the opposite side [11]. This syndrome is characterized by two separate infarctions as follows: one occurring at the junction of the left thalamomesencephalon and another in the left infratentorial paramedian area of the rostral midbrain.

Neuroimaging plays a crucial role in such cases, especially when multiple pathologies are evident. While a prospective study suggests that performing MRI on every patient older than 50 years with isolated cranial nerve III, IV, or VI palsy may not always be necessary, the young age of this patient with multiple pathological presentations warrants prompt neuroimaging to rule out life-threatening vascular events such as aneurysms or vascular malformations [12].

Another case report underscores the importance of neuroimaging even in cases of isolated abducens nerve palsy, as CT angiography revealed a ruptured posterior inferior cerebellar artery (PICA) aneurysm and subarachnoid hemorrhage [13]. A retrospective study establishes a localizing relationship between isolated abducens nerve palsy, subarachnoid hemorrhage, and an underlying ruptured PICA aneurysm [14]. Hence, isolated abducens nerve palsy should not be overlooked, and appropriate neuroimaging should be considered.

Moreover, an observational study demonstrated that rapid mechanical thrombectomy with successful reperfusion of the lenticulostriate arteries can protect the internal capsule from subsequent ischemia [15]. However, this approach was not applicable in the present case due to the timing of the presentation.

## Conclusions

Non-communicable diseases, including hypertension, diabetes mellitus, and hyperlipidemia, represent significant risk factors for stroke. Effective management and control of these conditions are essential to prevent complications such as ocular manifestations and potentially life-threatening cerebrovascular events.
